# Age-related alterations in retinal neurovascular and inflammatory transcripts

**Published:** 2011-05-07

**Authors:** Colleen A. Van Kirk, Heather D. VanGuilder, Megan Young, Julie A. Farley, William E. Sonntag, Willard M. Freeman

**Affiliations:** 1 Department of Pharmacology, Penn State College of Medicine, University Drive, Hershey, PA; 2 Donald W. Reynolds Department of Geriatric Medicine, University of Oklahoma Health Science Center, Oklahoma City, OK

## Abstract

**Purpose:**

Vision loss is one of the most common complications of aging, even in individuals with no diagnosed ocular disease. Increasing age induces structural alterations and functional impairments in retinal neurons and microvasculature linked to the activation of proinflammatory signaling pathways. Commonalities between the effects of aging and those observed with diabetes, including visual impairment, vascular dysfunction, and increased inflammatory response, have led to the hypothesis that diabetes-associated pathologies reflect an “advanced aging” phenotype. The goal of this study was to investigate the effects of aging on retinal mRNA expression of neurovascular and inflammatory transcripts previously demonstrated to be regulated with diabetes.

**Methods:**

The relative expression of 36 genes of interest previously identified as consistently regulated with diabetes was assessed in retinas of Young (3 month), Adult (12 month), and Aged (26 month) Fischer 344 x Brown Norway (F1) hybrid rats using quantitative PCR. Serum samples obtained at sacrifice were assayed to determine serum glucose levels.

**Results:**

Eleven inflammation- and microvascular-related genes previously demonstrated to be upregulated in young diabetic rats (complement component 1 s subcomponent [*C1s*], chitinase 3-like 1 [*Chi3L1*], endothelin 2 [*Edn2*], guanylate nucleotide binding protein 2 [*Gbp2*], glial fibrillary acidic protein [*Gfap*], intracellular adhesion molecule 1 [*Icam1*], janus kinase 3 [*Jak3*], lipopolysaccharide-induced TNF factor [*Litaf*], complement 1-inhibitor [*Serping1*], signal transducer and activator of transcription 3 [*Stat3*], tumor necrosis factor receptor subfamily member 12a [*Tnfrsf12a*]) demonstrated progressively increasing retinal expression in aged normoglycemic rats. Additionally, two neuronal function–related genes (glutamate receptor ionotropic NMDA 2A [Grin2a] and polycomb group ring finger 1 [*Pcgf1*]) and one inflammation-related gene (pigment epithelium-derived growth factor [*Pedf]*) displayed patterns of expression dissimilar to that previously demonstrated with diabetes.

**Conclusions:**

The commonalities in retinal age-related and diabetes-induced molecular alterations provide support for the hypothesis that diabetes and aging engage some common para-inflammatory processes. However, these results also demonstrate that while the retinal genomic response to diabetes and aging share commonalities, they are not superimposable phenotypes. The observed changes in retinal gene expression provide further evidence of retinal alterations in neurovascular and inflammatory processes across the adult rat lifespan; this is indicative of para-inflammation that may contribute to the functional impairments that occur with advanced age. The data also suggest the potential for an additive effect of aging and diabetes in the development of diabetic complications.

## Introduction

Even in the absence of ocular disease, deteriorating visual function is one of the most common complications of aging [1,2]. Numerous aspects of vision, ranging from general acuity to night vision, decline with advancing age, suggesting broad retinal dysfunction [3,4]. For example, contrast sensitivity (spatial resolution) decreases at around 50 years of age in humans, and deficits of temporal processing (motion sensitivity) are evident by 60 years [5]. This effect is exacerbated by low-light (scotopic) conditions [6] and likely reflects retinal dysfunction rather than decreased retinal illuminance, since age-related decreases in pupil diameter actually appear to improve contrast sensitivity [7]. Rod-mediated scotopic vision is impaired sooner and to a greater extent than photopic vision, and recovery latency after photobleaching is significantly increased with aging [8]. Importantly, these visual deficits occur even in subjects without age-related pathology (e.g., maculopathy, glaucoma) and with good acuity, and worsen progressively with increasing age [4,6-8]. Neurophysiological mechanisms for age-related vision impairment are reflected by altered electrophysiological responses of photoreceptors, bipolar cells, and ganglion cells in the peripheral retina and fovea. These cells, which mediate signal detection, integration, and output, exhibit increased response latency, decreased potential amplitude, and slowed temporal adaptation [2,9-13], suggesting decreased sensitivity and signal transduction with aging in the neural retina.

The retinal microvasculature also undergoes age-related changes, including altered ocular blood flow and loss of barrier integrity [14]. In clinically healthy adults with no potentially confounding cardiovascular symptoms, regulative retinal arterial constriction decreases drastically with aging, and may reflect the functional outcome of dysregulated cellular and biochemical barrier composition [15]. Retinal pigment epithelium (RPE) cells play integral roles in neuroretinal adhesion and metabolic support, as well as photoreceptor outer segment phagocytosis. As such, they are susceptible to the accumulation of photoreactive molecules, oxidized pigments, and toxic drusen deposits, and subsequent inflammatory response [16-19]. Indeed, age-related impairment of the blood-retina barrier has been described in aged rats, which exhibit significantly increased vascular permeability, decreased tight junction protein expression, and activation of major histocompatibility complex (MHC) class II–positive and phagocytic microglia compared to their younger adult counterparts [20]. The relationship between impaired retinovascular integrity and retinal inflammation is further demonstrated by oxidation/inflammation-induced RPE lesions and drusen deposition in a mouse model of age-related retinal degeneration [21]. Age-related retinal vascular lesions, specifically in the form of acellular capillaries, pericyte loss, and terminal deoxynucleotidyl transferase dUTP nick end labeling (TUNEL)-positive apoptotic endothelial cells, are highly similar to diabetes-related retinal pathology in the rat, and appear to develop at an accelerated rate with diabetes compared to normal aging [22]. Recent findings from Swaroop and colleagues integrate these processes in a cell-specific analysis examining the rod photoreceptor transcriptome with aging [23]. Altered oxidative phosphorylation, barrier integrity, and inflammatory homeostasis were observed in aged (12 month) mice.

The similarities between these complications and common effects of aging, particularly with regard to functional deficits of the central nervous system (CNS), have led to the hypothesis that diabetes contributes to the development of an “advanced aging” phenotype [24-27]. Diabetes mellitus is a metabolic disorder characterized by loss of insulin production or efficacy and glycemic dysregulation. There are currently more than 20 million people in the United States, nearly 8% of the population, living with diabetes and susceptible to its associated complications. Type 1 (insulin-dependent) diabetes, induced by peripubertal autoimmune destruction of insulin-producing pancreatic β-cells, comprises approximately 10% of diabetes cases worldwide [28], with the rest attributed to Type 2 (non-insulin-dependent) diabetes. Although the etiologies of Type 1 and Type 2 diabetes differ, the pathophysiology of these diseases is quite comparable, leading over time to complications that include nephropathy, peripheral neuropathy, cognitive decline, and retinopathy [28-33]. Although commonalities between aging and diabetes have been investigated in the cortex and hippocampus, few reports have focused on the retina, which is highly susceptible to homeostatic dysregulation.

Type 1 diabetes leads to some degree of diabetic retinopathy (DR) in approximately 95% of patients within 20 years of diagnosis, and although increasing awareness and improved glycemic control are decreasing the projected prevalence and severity of DR, it remains a leading cause of new cases of adult blindness worldwide [28,34,35]. Symptoms of DR include early neuronal dysfunction followed by neuronal apoptosis, vascular permeability, and upregulation of inflammatory signaling molecules. Visual impairment is associated with both manifest and subclinical DR, and includes deficits of hue discrimination and contrast sensitivity, delayed dark adaptation, abnormal visual fields, and decreased overall visual acuity [36-40]. Spatial resolution, or contrast sensitivity, is one of the most commonly studied aspects of vision consistently altered by diabetes. Early research in the field of retinopathy has demonstrated a generalized loss of central vision and hue discrimination, as well as significant decreases in both static and dynamic spatial resolution [41,42]. Many of these symptoms are common in aging individuals without diabetes or with other ocular conditions such as age-related macular degeneration or glaucoma, suggesting a progressive age-related visual impairment similar to that which occurs with DR. These similarities extend from the functional to the cellular level, as alterations in ocular blood flow, RPE integrity, microvasculature, Bruch’s membrane morphology, and electrophysiological correlates of neuronal function have all been reported with both DR and advanced aging.

Research using rat models of diabetes have contributed to the concept of DR as a progressive neurovascular complication that includes vascular, inflammatory, and neuronal components [43-47]. We have extensively characterized retinal transcriptomic and proteomic changes with diabetes [45,46,48,49], and observed induction of proinflammatory processes similar to those proposed with aging [50]. These findings support the hypothesis that DR represents an “advanced aging” phenotype; however, direct comparisons of the retinal molecular phenotypes with DR and aging are limited. To test the hypothesis that DR represents an advanced aging molecular phenotype, we assessed the retinal expression of several diabetes-responsive genes across the lifespan in a commonly employed rat model of aging, at young-adulthood (3 months), adulthood (12 months), and advanced age (26 months). We report both similarities and differences between young-adult diabetic and aged rat retinal gene expression patterns of the inflammatory, vascular, and neuronal processes. This analysis provides novel insight into the molecular changes that occur in the retina with increasing age, and demonstrates that advanced age and DR have common para-inflammatory processes.

## Methods

### Animals

Pathogen-free Fischer 344 x Brown Norway (F1) hybrid male rats, aged 3 months (Young-Adult), 12 months (Adult), and 26 months (Aged; n=5 per group) were obtained from the National Institute on Aging colony at Harlan Industries (Indianapolis, IN), as described previously [51]. All animals were singly housed in laminar flow cages (Polysulfone) in the Oklahoma University Health Sciences Center (OUHSC) Barrier Facility with free access to food and water (Purina Mills, Richmond, IN). The animal rooms were maintained on a 12h:12h light-dark cycle at constant temperature and humidity. The OUHSC animal facilities are fully accredited by the Association for Assessment and Accreditation of Laboratory Animal Care. All animal procedures were approved by the Institutional Animal Care and Use Committee in accordance with the Public Health Service Policy on Humane Care and Use of Laboratory Animals and the National Research Council’s Guide for the Care and Use of Laboratory Animals. Animals were sacrificed by decapitation without anesthesia and the retinas rapidly dissected, snap frozen in liquid nitrogen, and stored at −80 °C. Trunk blood was also collected at the time of sacrifice. At sacrifice, gross necropsy was performed and no visible tumors were evident in any of the animals. Visual inspection of the eye did not reveal cataracts in any of the animals.

### Serum glucose quantitation

Trunk blood was collected into 15 ml conical tubes (VWR, West Chester, PA) and separated by centrifugation at 2,500 rpm for 20 min at 4 °C. Following serum isolation (upper phase), samples were stored at −80 °C until analysis. Serum samples were analyzed for glucose levels by colorimetric assay (Glucose Assay Kit; Abcam, Cambridge, MA). Each sample was analyzed in technical triplicates with a standard curve ranging from 0 to 20 nmol/μl using a SpectraMax M2 microplate reader (Molecular Devices, Sunnyvale, CA) at 570 nm absorbance.

### RNA isolation and quantitation

Total RNA was isolated from snap-frozen retinas using standard extraction, precipitation, and purification methods, as previously described [45-48]. Briefly, retinas were homogenized in ice-cold Tri-Reagent (Sigma-Aldrich, St. Louis, MO) using an automated Retsch TissueLyser II bead mill (Qiagen, Inc., Germantown, MD) and stainless steel beads at 15 Hz for 1 min. 1-bromo-3-chloropropane (BCP; Molecular Research Center, Inc., Cincinnati, OH) was added in 1/10th volume to separate phases. Isopropanol was then added at 1/10th volume to precipitate RNA from the aqueous phase. Precipitated RNA was purified using Qiagen RNeasy spin columns (Qiagen, Inc., Valencia, CA) and subsequently resuspended in RNase-free water. Quality and quantity were evaluated using the RNA 6000 Nano LabChip with an Agilent 2100 Expert Bioanalyzer (Agilent, Palo Alto, CA) and NanoDrop ND100 (Nanodrop, Wilmington, DE), respectively. RNA quality, as measured by a Bioanalyzer, was determined using RNA integrity numbers, which are a measure of RNA degradation ranging from 10 (intact) to 2 (degraded). RNA purity and concentration, as measured by a NanoDrop spectrophotometer, was determined by absorbance at 260 and 280 nm.

### Quantitative reverse transcription PCR

Following RNA isolation and quality control, cDNA was synthesized using the ABI High Capacity cDNA Reverse Transcription Kit (Applied Biosystems Inc., Foster City, CA) according to standard protocols [46,49]. Briefly, 2× reverse transcription (RT) master mix was prepared by mixing 2 μl 10× RT Buffer, 0.8 μl 25× 100 mM deoxynucleotide triphosphates (dNTPs), 2 μl 10× random primers, 1 μl MultiScribe Reverse Transcriptase, 1 μl RNase Inhibitor, and 3.2 μl nuclease-free water per reaction (total of 15 reactions) for a total of 10 μl master mix per reaction. Total RNA (1 μg/10 ul) was pipetted into each well followed by 10 μl of the prepared 2× master mix. Reverse transcription was performed using a GeneAmp PCR System 9700 (Applied Biosystems Inc.) with the following settings: Step 1) 25 °C – 10 min; 2) 37 °C – 2 h; 3) 85 °C – 5 s; 4) hold at 4 °C.

Quantitative PCR analysis was performed using a 7900HT Sequence Detection System (Applied Biosystems Inc.), 384-well optical plates, and Assay-On-Demand gene expression assays containing the gene-specific primers and probes listed in Table 1 (Applied Biosystems Inc.) [46,49]. All samples were assayed in technical triplicates. Briefly, the PCR reaction mix was prepared by mixing 0.125 μl 20× gene expression assay and 5 μl 2× Gene Expression Master Mix (Applied Biosystems Inc.) per reaction. Then, 50 ng cDNA (5 μl of 10 ng/μl stock solution) was pipetted into each well, followed by 5 μl of the prepared master mix, yielding a 10 μl reaction. The prepared plate was incubated at 50 °C for 2 min and at 95 °C for 10 min, followed by 40 PCR with the following specifications: 1) 95 °C – 15 s; 2) 60 °C – 1 min. Relative amounts of gene transcript were determined using SDS 2.2.2 software (Applied Biosystems Inc.) and the 2^-ΔΔCt^ method with β-actin (*Actb*) as the endogenous control [52,53].

**Table 1:** Gene names, ID numbers, and qPCR expression assays.

**Gene symbol**	**GeneID number**	**Gene name**	**Gene expression assay catalog #**
*Bbs2*	113948	Bardet - Biedl syndrome 2	Rn00586096_m1
*Birc4*	63879	X-linked inhibitor of apoptosis	Rn00573706_m1
*C1S*	192262	Complement component 1, s subcomponent	Rn00594278_m1
*Carhsp1*	260416	Calcium regulated heat stable protein 1	Rn00596083_m1
*Ccr5*	117029	Chemokine, CC Motif, Receptor 5	Rn00588629_m1
*Chi3L1*	89824	Chitinase 3-like 1	Rn01490608_m1
*Dcamkl1*	83825	Doublecortin-like kinase 1	Rn00584294_m1
*Edn2*	24324	Endothelin 2	Rn00561135_m1
*Ednrb*	50672	Endothelin receptor B	Rn00569139_m1
*Elovl1*	679532	Elongation of very long chain fatty acids	Rn01403757_m1
*Eno2*	24334	Enolase 2	Rn00595017_m1
*Gat3*	79213	GABA transporter 3	Rn00577664_m1
*Gbp2*	171164	Guanylate nucleotide binding protein 2	Rn00592467_m1
*Gfap*	24387	Glial fibrillary acidic protein	Rn00566603_m1
*Grin2a*	24409	Glutamate receptor, ionotropic, NMDA 2A	Rn00561341_m1
*Hspb1*	24471	Heat shock 27 kDa protein 1	Rn00583001_g1
*Icam1*	25464	Intracellular adhesion molecule 1	Rn00564227_m1
*Igf2*	24483	Insulin - like growth factor 2	Rn00580426_m1
*Jak3*	25326	Janus Kinase 3	Rn00563431_m1
*Kcne2*	171138	Isk-related voltage-gated K+ channel 2	Rn02094913_s1
*Lama5*	140433	Laminin, alpha 5	Rn01415966_g1
*Lgals3*	83781	Lectin, galactoside-binding, soluble, 3	Rn00582910_m1
*Lgals3bp*	245955	Lectin, galactoside-binding, soluble, 3, binding protein	Rn00478303_m1
*Litaf*	65161	Lipopolysaccharide-induced TNF factor	Rn01424675_m1
*Mccc1*	294972	Methylcrotonoyl-CoA carboxylase 1	Rn01462252_m1
*Nefh*	24587	Neurofilament, heavy polypeptide	Rn00709325_m1
*Nppa*	24602	Natriuretic peptide precursor A	Rn00561661_m1
*Nr3c1*	24413	Nuclear receptor subfamily 3, group C, member 1	Rn01405584_m1
*Pcgf*	312480	Polycomb group ring finger 1	Rn01425394_g1
*Pedf*	287526	Pigment epithelium-derived growth factor	Rn00709999_m1
*Serping1*	295703	Complement 1 - inhibitor	Rn01485600_m1
*Stat1*	25124	Signal transducer and activator of transcription 1	Rn00583505_m1
*Stat3*	25125	Signal transducer and activator of transcription 3	Rn00562562_m1
*Timp1*	116510	Tissue inhibitor of metalloproteinase 1	Rn00587558_m1
*Tnfrsf12*	302965	Tumor necrosis factor receptor subfamily, member 12a	Rn00710373_m1
*Vegfa*	83785	Vascular endothelial growth factor A	Rn00582935_m1

### Statistical analyses

Serum glucose concentrations and transcript expression data were analyzed by one-way ANOVA (ANOVA) with Student–Newman–Keuls (SNK) post-hoc testing with α<0.05. For the gene expression analysis, a Benjamini-Hochberg multiple testing correction was applied to the one-way ANOVA across the 36 genes examined to control for Type I false positive errors [54]. For any data that failed normality testing, a Kruskal–Wallis one-way ANOVA on ranks was performed using SigmaStat 3.5 (Systat Software, San Jose, CA). Potential overrepresentation of genes commonly regulated with aging and diabetes was tested using a binomial distribution, assuming the independence of each gene and equal distribution of increases and decreases in gene expression by random chance. Principal component analysis was used to visualize the relationship between groups. Individual animal data for Young, Adult, and Aged rats from all 36 genes quantitated were normalized to mean Young values and imported into GeneSpring GX 11.0. Likewise, previously reported expression data for 3-month diabetic and nondiabetic control rats [45,46,49] were scaled to mean control values for comparison with age-group data. The first two principal components were mean centered and scaled, and plotted with individual animal data compressed to represent each group as a single point. Potential common transcriptional regulators were assessed by Ingenuity Pathway Analysis (Ingenuity Systems, Redwood City, CA).

## Results

### Serum glucose

**Figure 1:**
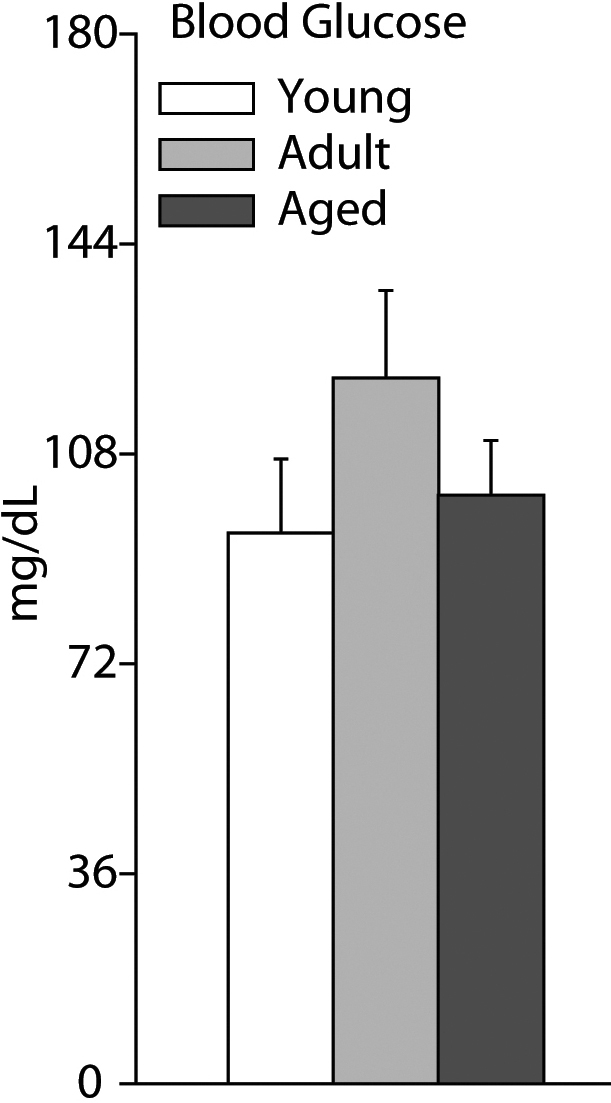
Serum glucose levels measured at sacrifice. Non-fasting serum glucose levels were measured at sacrifice. All glucose levels were normoglycemic and no differences between groups were observed. One-way ANOVA (ANOVA) with Student–Newman–Keuls (SNK) post hoc test, n=5–6/group. Error bars represent standard error of the mean.

Serum glucose levels at sacrifice were assessed to confirm normoglycemia. The average serum glucose level in Young animals was 5.24 nM±0.7 (94 mg/dl), 6.72 nM±0.8 (121 mg/dl) in Adult animals, and 5.60 nM±0.5 (101 mg/dl) in Old animals (Figure 1). All groups were normoglycemic and no statistically significant differences were found between the groups. No individual animals were hyperglycemic (>250 mg/dl).

### Retinal mRNA expression

To evaluate potential similarities between retinal transcript expression changes with aging and diabetes, 36 gene targets from our previously described analyses of DR [45-48] were quantitated in Young, Adult, and Aged rats by quantitative PCR. Our previous analyses of retinal gene expression with diabetes have shown that these transcripts are differentially expressed in a highly consistent manner across multiple experiments [46]. Of the 36 genes examined, 14 were differentially expressed (one-way ANOVA, Benjamini-Hochberg multiple testing correction, p<0.05). Eleven genes were increased similarly to what we have previously demonstrated with diabetes. Three genes were regulated in a direction opposite to that seen in diabetes. More genes were regulated in the same direction and magnitude with aging and diabetes than would be expected by chance (p=0.028), assuming a random distribution. To test for the potential coregulation of these genes, Ingenuity Pathway Analysis was performed and no transcription factors or other transcriptional regulators were identified as common to more than three of the differentially expressed genes. However, more complex regulatory networks that are not currently understood may be commonly engaged by aging and diabetes, which would affect the assumption of a random distribution. Although these genes have multiple functions, they can be separated into three distinct groups according to our previous analyses [45]: inflammation-related genes, microvascular-related genes, and neuronal function–related genes.

### Inflammatory transcripts

**Figure 2:**
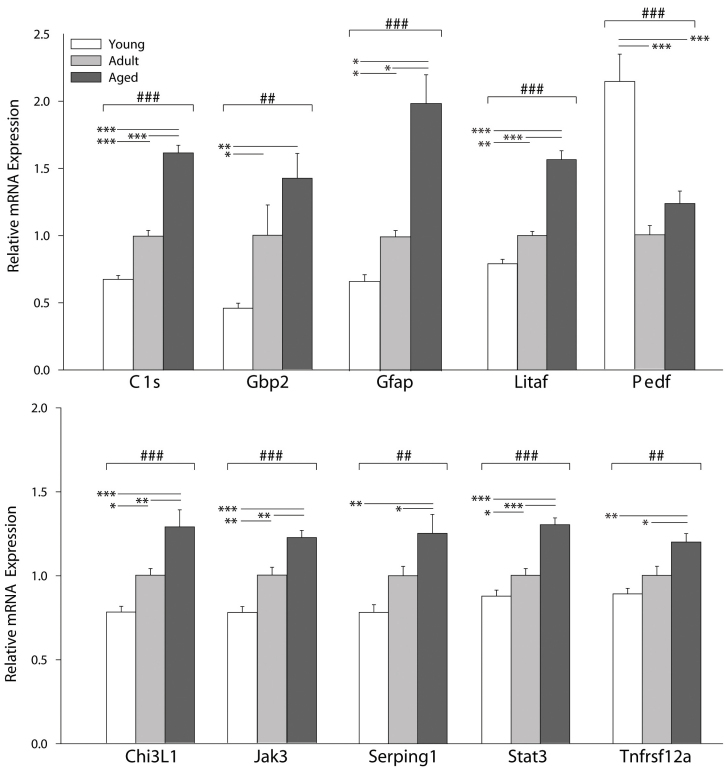
Inflammation transcripts are regulated with aging. Ten of the 23 inflammation-related transcripts that we have previously observed to be upregulated in the retina of a rat model of Type I diabetes were significantly altered with age. With the exception of pigment epithelium-derived growth factor (*Pedf*), all of the transcripts were upregulated in Old animals. One-way ANOVA (ANOVA) with Benjamini-Hochberg multiple testing correction, ##p<0.01, ###p<0.001; SNK post-hoc test, *p<0.05, **p<0.01, ***p<0.001, n=5/group. Error bars represent standard error of the mean.

For the genes associated with inflammatory processes, most of the changes were upregulations with age. Retinal transcript expression of complement component 1 s subcomponent (*C1s*), glial fibrillary acidic protein (*Gfap*), lipopolysaccharide-induced TNF factor (*Litaf*), chitinase 3-like 1 (*Chi3L1*), janus kinase 3 (*Jak3*), and signal transducer and activator of transcription 3 (*Stat3*) was significantly higher in Adult animals when compared to Young animals. Additionally, these transcripts showed significantly higher expression in Aged versus both Young and Adult animals (Figure 2). Complement 1-inhibitor I (*Serping1*) and tumor necrosis factor receptor subfamily member 12a (*Tnfrsf12a*) demonstrated a similar pattern of expression with significantly higher expression levels in Aged versus both Young and Adult animals. Guanylate nucleotide binding protein 2 (*Gbp2*) expression was significantly elevated in Aged and Adult versus Young animals. Pigment epithelium-derived growth factor (*Pedf*) showed significantly lower expression levels in both Adult and Aged animals compared to the Young group. Additional genes examined (X-linked inhibitor of apoptosis [*Birc4*], calcium regulated heat stable protein 1 [*Carhsp1*], chemokine CC motif receptor 5 [*Ccr5*], elongation of very long chain fatty acids [*Elovl1*], heat shock 27 kDa protein 1 [*Hspb1*], lectin galactoside-binding soluble 3 [*Lgals3*], lectin galactoside-binding soluble 3 binding protein [*Lgals3bp*], methylcrotonoyl-CoA carboxylase 1 [*Mccc1*], nuclear receptor subfamily 3 group C member 1 [*Nr3c1*], signal transducer and activator of transcription 1 [*Stat1*], and tissue inhibitor of metalloproteinase 1 [*Timp1*]) did not differ in expression between groups (Table 2). With the exception of *Pedf*, all of the age-related gene expression changes were induced in a similar fashion to what was seen in diabetes (Table 3).

**Table 2:** Transcripts not regulated with age.

**Gene**	**Young**	**Adult**	**Old**	**Function**
*Birc4*	1.0±0.05	1.0±0.03	0.9±0.03	Inflammation
*Carhsp1*	0.9±0.07	1.0± 0.13	1.0±0.17	
*Ccr5*	1.0±0.03	1.0± 0.04	1.0±0.03	
*Elovl1*	1.0±0.06	1.0± 0.04	0.9±0.04	
*Hspb1*	0.9±0.13	1.0± 0.06	1.1±0.08	
*Lama5*	1.1±0.06	1.0± 0.06	1.1±0.07	
*Lgals3*	1.1±0.08	1.0± 0.05	1.1±0.08	
*Lgals3bp*	1.0±0.05	1.0±0.03	0.9±0.03	
*Mccc1*	1.0±0.05	1.0± 0.04	1.0±0.04	
*Nr3c1*	0.9±0.06	1.0± 0.02	1.1±0.05	
*Stat1*	1.0±0.04	1.0± 0.06	1.2±0.07	
*Timp1*	1.0±0.03	1.0± 0.06	1.1±0.09	
*Ednrb*	1.0±0.05	1.0± 0.06	1.0±0.08	Microvasculature
*Nppa*	0.9±0.02	1.0±0.16	1.1±0.06	
*Vegfa*	1.0±0.08	1.0±0.03	1.1±0.06	
*Bbs2*	1.0±0.05	1.0±0.03	1.0±0.05	Neuronal function
*Dcamkl1*	1.0±0.05	1.0± 0.11	1.0±0.06	
*Eno2*	1.0±0.05	1.0± 0.02	1.0±0.04	
*Gat3*	1.0±0.05	1.0± 0.05	1.0±0.05	
*Igf2*	0.8±0.05	1.0± 0.05	1.0±0.04	
*Kcne2*	1.1±0.08	1.0± 0.09	0.9±0.07	
*Nefh*	1.0±0.04	1.0±0.03	1.1±0.05	

Data are presented as mean±SEM (n=5/group) for genes which were not significantly regulated with age (Benjamini Hochberg multiple testing corrected one-way ANOVA with Student–Newman–Keuls (SNK) post-hoc testing or Kruskal–Wallis One-Way ANOVA on Ranks if the data failed normality testing).

**Table 3:** Genes previously characterized in rat retina with diabetes.

**Gene**	**Diabetic/Control**	**p-value**
*Bbs2*	↓ 40%	***
*Birc4*	↓ 30%	***
*C1-INH*	↑ 160%	**
*C1S*	↑ 180%	**
*Carhsp1*	↑ 160%	***
*Ccr5*	↑ 60%	**
*Chi3L1*	↑ 170%	**
*Dcamkl1*	↓ 20%	**
*Edn2*	↑ 270%	*
*Ednrb*	↑ 60%	**
*Elovl1*	↓ 50%	***
*Eno2*	↓ 20%	*
*Gat3*	↓ 40%	***
*Gbp2*	↑ 140%	*
*Gfap*	↑ 400%	**
*Grin2a*	↓ 40%	***
*Hspb1*	↑ 1320%	**
*Icam1*	↑ 70%	**
*Igf2*	↓ 50%	***
*Jak3*	↑ 90%	**
*Kcne2*	↓ 50%	***
*Lama5*	↑ 80%	***
*Lgals3*	↑ 700%	***
*Lgals3bp*	↑ 70%	**
*Litaf*	↑ 70%	*
*Mccc1*	↓ 20%	**
*Nefh*	↓ 20%	*
*Nr3c1*	↓ 20%	*
*Nppa*	↓ 50%	***
*Pcgf1*	↓ 30%	*
*Pedf*	↑ 90%	*
*Stat1*	↑ 30%	*
*Stat3*	↑ 40%	**
*Timp1*	↑ 300%	**
*Tnfrsf12a*	↑ 120%	***
*Vegfa*	↓ 40%	***

Data represent gene expression in the diabetic rat retina as a percent of control values. Two-tailed *t*-test *p<0.05, ** p<0.01, *** p<0.001. From [44,45,47].

### Microvascular transcripts

**Figure 3:**
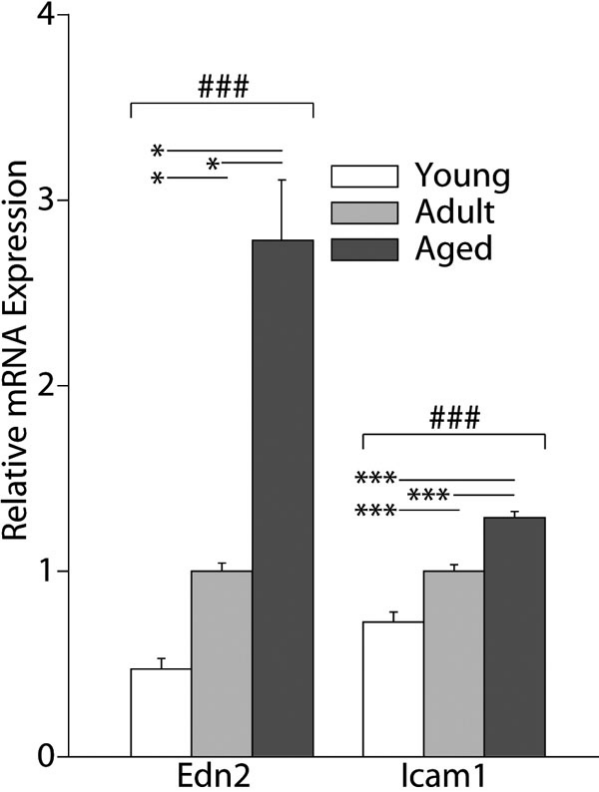
Microvascular transcripts are regulated with aging. Two of the five microvascular-related transcripts that we have previously observed to be upregulated in the retina of a rat model of Type I diabetes were significantly altered with age. One-way ANOVA (ANOVA) with Benjamini-Hochberg multiple testing correction, ###p<0.001; SNK post-hoc test, *p<0.05, **p<0.01, ***p<0.001, n=5/group. Error bars represent standard error of the mean.

Of the microvascular-related transcripts measured, endothelin 2 (*Edn2*) and intracellular adhesion molecule 1 (*Icam1*) were both found to significantly increase in expression in the retina with age (Figure 3). In particular, *Edn2* showed significantly higher expression levels in the Aged versus Young and Adult rats, as well as in Adult versus Young rats (Kruskal–Wallis one-way ANOVA on ranks, p<0.05). Similarly, *Icam1* showed significantly higher expression in Aged versus Young and Adult versus Young animal comparisons (SNK, p<0.001). No differences in expression of endothelin receptor B (*Ednrb*), natriuretic peptide precursor A (*Nppa*), or vascular endothelial growth factor A (*Vegfa*) were observed between age groups (Table 2). Similar to the age-related gene expression changes, both *Edn2* and *Icam1* showed significantly higher expression in rats with diabetes compared to nondiabetic controls (Table 3).

### Neuronal function transcripts

**Figure 4:**
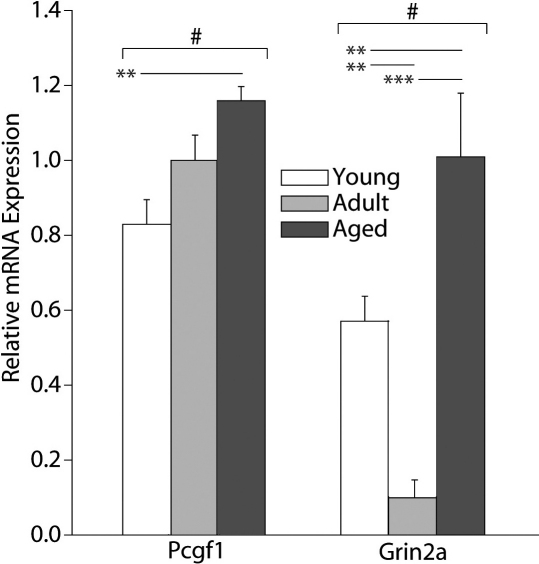
Neuronal function transcripts are regulated with aging. Two of the 10 neuronal function–related transcripts that we have previously observed to be upregulated in the retina of a rat model of Type I diabetes were significantly altered with age. Polycomb group ring finger 1 (*Pcgf*) increased with age while glutamate receptor ionotropic NMDA 2A (*Grin2a*) decreased in Adult animals and increased in the Aged animals. One-way ANOVA (ANOVA) with Benjamini-Hochberg multiple testing correction, #p<0.05; SNK post-hoc test, *p<0.05, **p<0.01, ***p<0.001, n=5/group. Error bars represent standard error of the mean.

Two genes associated with neuronal function were also found to be significantly regulated with age in the rat retina (Figure 4). Polycomb group ring finger 1 (*Pcgf1*) demonstrated significantly higher transcript levels when comparing Aged rats to Young ones (SNK, p<0.005). Glutamate receptor ionotropic NMDA 2A (*Grin2a*) also showed significantly higher levels of gene expression in Aged than Young rats (Student–Newman–Keuls [SNK], p=0.007) or Adult animals (SNK, p<0.001). However, *Grin2a* levels significantly decreased in Young as compared to Adult animals (SNK, p=0.002). Bardet-Biedl syndrome 2 (*Bbs2*), doublecortin-like kinase 1 (*Dcamkl1*), enolase 2 (*Eno2*), GABA transporter 3 (*Gat3*), isk-related voltage-gated K+ channel 2 (*Kcne2*), and neurofilament heavy polypeptide (*Nefh*) showed no significant differences between age groups (Table 2). In contrast to these results, *Pcgf1* and *Grin2a* showed significantly decreased expression with diabetes as compared to nondiabetic controls (Table 3).

**Figure 5:**
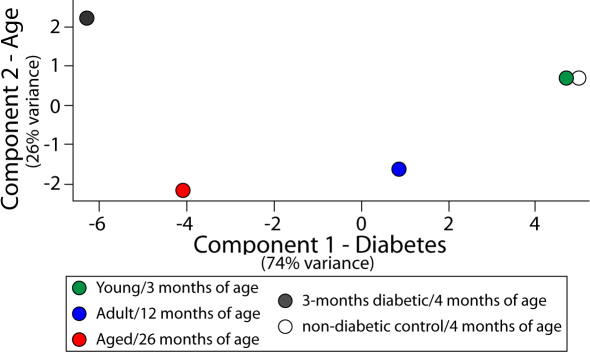
Principal component analysis demonstrates relationship between patterns of aging- and diabetes-induced retinal gene expression. To visualize the relationship of gene expression profiles across age groups, a principal component analysis was performed using genes determined to be significantly regulated with aging. A clear progression in expression profiles was evident with increasing age, and created a continuum ranging from the Young to Adult to Aged group. Previously reported expression data for 3-month diabetic and nondiabetic control rats, aged 4 months, were included in this analysis. Diabetic rats were separated from controls in a similar manner to the Aged group, demonstrating commonalities in expression changes and lending support to the hypothesis of diabetes as a form of accelerated aging.

To visualize the relationship of gene expression profiles between age groups and our previously published characterizations of the rat retina after three months of diabetes, a principal component analysis was performed. The principal component analysis was used to combine all of the data into one visualization showing the relationship of the expression patterns from the different groups. For the purposes of this study, each gene was plotted on its own axis to form a multidimensional space from which the principal components were identified in order of decreasing variance. This permits visualization of the most significant relationships between groups in a standard two-dimensional plot, when the first two components are plotted. Retinal gene expression data was scaled to the mean of the Young group for each gene. Data from diabetic and nondiabetic controls were scaled to the mean of the controls on a gene-by-gene basis. The first component accounted for 74% of variance between groups, progressively separating the Young and Nondiabetic Control from the Adult, Aged, and Diabetic rats along the x-axis (Figure 5); this was interpreted as gene expression changes with diabetes. The second component (26% of variance) separated the Young, Nondiabetic Control, and Diabetic groups from the Adult and Aged groups along the y-axis. This component was interpreted as representing the age-specific changes in gene expression. In total, this visualization demonstrates that while there are some commonalities in gene expression patterns with diabetes and aging, they are not superimposable molecular phenotypes.

## Discussion

Aging is a primary risk factor for vision loss. With increasing age, functional and structural characteristics of the retina decline, as is demonstrated by aberrant psychophysical and electrophysiological measures of stimulus response, impairment of vascular integrity, and activation of inflammatory signaling. Numerous aspects of retinal dysfunction with normal aging share commonalities with pathological changes and functional deficits induced with diabetes [10,22,50,55,56]. Commonalities between aging and diabetes have been reported in both the CNS and the periphery, and have led to the hypothesis that diabetes may induce an “accelerated aging” phenotype. The goal of this study was to characterize age-related retinal expression profiles of neurovascular and inflammatory transcripts, using targets previously determined to be regulated with diabetes. Many of the transcripts investigated exhibited progressively increased expression with increasing age, in agreement with functional data indicating generalized age-related neurovascular inflammation in the retina and consistent with the concept of para-inflammation [57]. The functions of these genes and their protein products, as well as the potential implications of their dysregulation with aging, are discussed below.

### Microvasculature

Previous investigations of microvascular dysfunction with retinal aging have demonstrated declining RPE cell integrity [58], microvascular lesions [56,59], increased vessel leakage, and vascular cell apoptosis [22]. In the aged retina, decreased occludin expression is associated with increased vascular permeability [20] and RPE dysfunction, although the specific biochemical mechanisms of declining vascular integrity remain to be fully determined. We have previously demonstrated that *Edn2*, *Ednrb*, *Icam1*, *Nppa*, and *Vegfa* showed significantly altered expression patterns in the diabetic rat retina when compared to nondiabetic controls [45]. *Icam1* and *Edn2* are increased with diabetes and show similar magnitude increases in expression with age. *Icam1* is elevated in patients diagnosed with proliferative DR, and contributes to blood-retinal breakdown and DR development [60-62]. Our findings agree with a previous report of increased retinal *Icam1* expression with advanced age [62]. *Icam1* has been implicated in leukocyte cell adhesion to the retinal vascular endothelium in the diabetic retina, promoting leukostasis and therefore retinal endothelial cell injury and death [19,63]. Increased *Icam1* expression with aging could work additively in aging diabetics to increase the risk of microvascular dysfunction. *Edn2* is a strong vasoconstrictor and regulator of ocular blood flow [64]. As blood flow abnormalities are observed with both diabetes and aging, the common upregulation of *Edn2* may represent a common causative factor. *Edn2* expression has been localized to photoreceptors and signals through the *Ednrb* receptor on Müller cells [65]. This signaling may serve as an integration point between microvascular dysfunction, microglial activation, and inflammation, which are observed in both diabetes and aging.

One interesting finding was that there was no change in *Vegf* with age in the retina. Previously, we have consistently identified a modest but significant decrease in *Vegf* mRNA in a streptozotocin (STZ)-induced rat model of diabetes [45,46]. While induction of retinal Vegf protein in late-stage DR patients has been described, data on the exact time course of *Vegf* induction are conflicting, with reports of *Vegf* expression being unchanged at 3 months [66], increased at 6 months [67], and decreased at 6 months [68] of STZ-induced diabetes in the rat.

### Neuronal function

Neuronal dysfunction and vision loss with aging are well characterized at the psychophysical, electrophysiological, and biochemical levels, and suggest broad impairment of the neural retina that affects photoreceptors, interneurons, and ganglion cells [9,10,13]. Our previous studies have shown marked alterations in the expression of *Bbs2*, *Dcamkl1*, *Eno2*, *Gat3*, *Grin2a*, *Kcne2*, *Nefh*, and *Pcgf1* in the diabetic retina (Table 3). Of these genes, two (*Pcgf1* and *Grin2a*) were found to be regulated with age. *Pcgf1* increased significantly with age while *Grin2a* showed decreased expression in Adults when compared to Young animals, yet Aged animals showed significantly increased expression when compared to both Young and Adult animals. These results are interesting, as both of these genes show a general upregulation with age while with diabetes, they were found to show significant downregulation.

*Pcgf1*, also known as *Nspc1* (nervous system polycomb 1), is a novel transcriptional repressor that is highly expressed in the developing nervous system. This gene has been demonstrated to be responsive to protein kinase C (PKC) signaling and has also been shown to be associated with epigenetic mechanisms of gene transcription regulation [69,70]. Upregulation of this gene in aged animals could alter transcriptional control of genes regulated by *Pcgf1*, either directly (functioning as a transcriptional repressor) or indirectly (functioning as an epigenetic modifier). Further gene-specific studies of *Pcgf1* are needed to determine the specific target genes regulated by *Pcgf1*.

*Grin2a* (glutamate receptor, ionotropic, N-methyl D-aspartate 2A) encodes the glutamatergic NMDAR2a receptor responsible for excitatory neurotransmission. Although the function of this receptor is unknown in the retina, the heteromeric subunit composition of the NMDA receptor is associated with variations in excitotoxicity [71]. *Grin2a* demonstrated a unique gene expression profile across ages, decreasing in adulthood as compared to Young animals, while seeming to overcompensate and show the highest expression in Aged animals. This could be a maladaptive compensatory response to other changes in neuronal gene expression and diminished neurotransmission that occur with advanced age, given the sharp increase in expression found specifically in the Aged animals. The functional outcome of increased *Grin2a* could facilitate neurotransmission, but also result in excitotoxicity and the induction of downstream inflammatory processes. The hypothesis seems to be plausible given the higher *Grin2a* expression in both the Young and Aged animals.

### Inflammation

Age-related increases in inflammatory processes are reported throughout the CNS. In the retina, vascular dysfunction likely contributes to proinflammatory signaling in a feed-forward cycle due to loss of barrier integrity, toxic drusen deposition, and monocyte infiltration [17,72]. Increased microglial activation, indicated by the presentation of MHC class II- and ED1-immunoreactive cells, has been reported in the retinal parenchyma in aged rats [20]. In aged mice (20 months), markers of para-inflammation observed in this study (*C1s, Gpb2, Icam1,* and *Spering1*) have been reported [62]. Furthermore, elevated circulating levels of the proinflammatory glycoprotein *Chi3L1* have been demonstrated in aged humans (>80 years) compared with younger adults (18–30 years) [19]. Oxidative stress, the accumulation of photoreactive pigments, necrotic/apoptotic cells, and leukocyte infiltration have been implicated in the activation of inflammatory pathways involving these and other proinflammatory molecules.

A hallmark of DR is a marked increase in the expression of genes involved in the regulation of inflammatory processes. We have previously demonstrated that *Birc4*, *Carhsp1*, *C1-Inh* (*Serping1*), *C1s*, *Ccr5*, *Cd44*, *Chi3L1*, *Elovl1*, *Gbp2*, *Gfap*, *Hspb1*, *Jak3*, *Lama5*, *Lgals3*, *Lgals3bp*, *Litaf*, *Mccc1*, *Nr3c1*, *Pedf*, *Stat1*, *Stat3*, *Timp1*, and *Tnfrsf12a* significantly change in expression in the diabetic retina. Of these genes, 10 were found to be regulated with aging, specifically *C1s*, *Chi3L1*, *Gbp2*, *Gfap*, *Jak3*, *Litaf*, *Pedf*, *Serping1*, *Stat3*, and *Tnfrsf12a*. All of these genes increased in expression with DR, and several have been previously reported to change with aging in the CNS. Interestingly, similar increases in expression were seen with age in all genes except *Pedf,* which was actually found to significantly decrease in expression.

*C1s* (complement component 1, s subcomponent) encodes a serine protease that is a subunit of the complement subcomponent C1 complex, which is involved in activation of the complement system. Although the exact role of *C1s* upregulation in DR and aging is unknown, it has been shown to be expressed in retinal ganglion cells. Interestingly, elevated *C1s* expression was observed with retinal ganglion cell death in response to serum depravation, suggesting a role for the complement pathway in regulating neuronal cell death in the retina [73]. Therefore, elevated expression of *C1s* could be indicative of complement pathway activation and increased inflammation in response to retinal ganglion cell death. Furthermore, *Chi3L1* (chitinase 3–like 1) is an inflammatory glycoprotein secreted by activated macrophages and is suggested to be involved in innate immune system activation, macrophage differentiation, extracellular matrix remodeling, and vascular cell migration [74]. Our tissue-specific findings agree with clinical reports of increased plasma Chi3L1 levels in Type 1 and Type 2 diabetic subjects [75,76]. Elevated *Chi3L1* expression in the retina implies a general increase in retinal inflammation with both diabetes and aging. Although the exact role of *Gbp2* (guanylate binding protein 2, interferon-inducible), an interferon-inducible GTPase, is unknown in the pathology of DR, our previous studies have shown a significant increase in expression in diabetic animals when compared to nondiabetic controls [46].

*Gfap* (glial fibrillary acidic protein) is an intermediate filament protein highly expressed in astrocytes that has been shown to be differentially expressed with DR. Increased *Gfap* expression in Müller cells with diabetes has been described previously [44,68], and we have observed the insulin therapy–resistant induction of *Gfap* mRNA with diabetes [49]. The step-wise induction of *Gfap* with increasing age is prototypical of increased para-inflammation with aging. *Jak3* (janus kinase 3) is a proinflammatory component of the Jak-Stat signaling pathway that activates *Stat3* (signal transducer and activator of transcription 3). This pathway regulates cytokine signaling and inflammatory responses [77]. Upregulation of *Jak3* and *Stat3* signaling with aging can induce a broad range of acute phase and other inflammatory mediators. The transcription factor *Litaf* (lipopolysaccharide-induced TNF factor) regulates the production of TNF-α, a potent and well characterized proinflammatory cytokine in the retina [78]. Our studies have demonstrated significant increase in *Litaf* expression with both DR and age, suggesting increased levels of inflammation. *Tnfrsf12a* (tumor necrosis factor receptor superfamily, member 12 A; aka Tweakr, Fn14) is the receptor for TNF-related weak inducer of apoptosis (Tweak) which is a proinflammatory cytokine. Previous studies have demonstrated that Tnfrsf12a activation via Tweak has proinflammatory effects in RPE cells [79] and produces more highly vascularized tumors in athymic mice [80] suggesting both proinflammatory and proangiogenic effects with increased expression.

Contrary to the genes described above, *Pedf* (pigment epithelium derived factor) has anti-inflammatory properties and has been found to be enriched in the serum of patients with Type 1 diabetes with DR [81]. In one study, *Pedf* was able to inhibit the migration of cultured endothelial cells in a dose-dependent manner, suggesting its ability to decrease neovascularization [82]. Additionally, a recent report showed how *Pedf* was able to decrease advanced glycation end-product (AGE)-induced endothelial cell permeability, further suggesting that *Pedf* plays a role in counteracting angiogenesis associated with DR [83], and AGE-induced inflammation with aging and diabetes [55]. Decreased *Pedf* expression in the adult and aged retina would diminish a critical protective mechanism against AGE/AGE receptor (RAGE)-induced inflammation, leaving the retina more susceptible to a variety of physiologic insults. *Serping 1* (serpin peptidase inhibitor, clade G [C1 inhibitor], member 1), also known as *C1-Inh*, is an inhibitor of the complement system activation. Patients with DR show increased expression of *Serping1*, which serves to protect the integrity of the vascular endothelium in the retina [84]. Unlike the diminished expression of *Pedf*, this protective mechanism is induced with age.

Taken together, the induction of proinflammatory genes (*C1s*, *Chi3L1*, *Gbp2*, *Gfap*, *Jak3*, *Litaf*, *Stat3*, and *Tnfrsf12a*) and reduction of the anti-inflammatory *Pedf* indicate a para-inflammatory state with increasing age in the retina [50]. This generalized proinflammatory state could help explain the increased vulnerability for retinal diseases such as DR and macular degeneration with increasing age. The potential mechanisms leading to the para-inflammatory state observed with increasing age include retinal stress through reactive oxygen species and AGEs. The commonalities in retinal gene expression changes occurring in young hyperglycemic and aged normoglycemic rats indicate that different stressors can lead to some, but not all, of the same inflammatory processes. Clearly, additional research is needed to build upon the existing knowledge related to retinal para-inflammation with aging [62].

These results demonstrate altered retinal gene expression across the adult rat lifespan in a set of genes previously demonstrated to be altered in expression with diabetes. While this analysis focused on a predetermined set of genes, these results provide the rationale for future genome-wide comparisons of retinal transcriptomic changes with aging and diabetes, as well as comparative analysis of retinal proteomic alterations. For both molecular and biochemical analyses, use of isolated retinal cell types, such as those previously reported (e.g., Müller [68], photoreceptors [23]), will provide a clearer picture of the altered pathways and processes with aging and diabetes in retinal cell types. While technically challenging, cell-specific analyses of in vivo models is needed to more clearly tie altered RNA/protein expression to dysfunction in specific cell types and to define gene expression regulatory networks that may be commonly engaged with aging and diabetes. Prospective studies across the lifespan, including induction of diabetes at different ages, will provide more specific insight into whether the effects of aging and diabetes are additive.

The specific inflammatory, neuronal function, and microvascular genes altered with aging provide potential molecular mechanisms for the well documented functional impairments and para-inflammatory state of the retina with advanced age. Additionally, these results support the hypothesis that diabetes and aging share some common molecular alterations. However, these results also demonstrate that the patterns of gene expression with advanced age and diabetes are not identical. Age-related changes in retinal gene expression indicative of para-inflammation may work synergistically with the same para-inflammatory processes induced by diabetes to accelerate DR pathogenesis. Given the growing incidence of diabetes and increasing longevity [85], further investigation of the contribution of age-related retinal gene expression alterations to retinal diseases common in the aged population is needed. The microvascular and inflammatory processes observed in this study may also serve as targets for development of therapies that prevent or treat age-related retinal dysfunction.

## References

[1] National Eye Institute. Prevalence of blindness and low vision among adults aged 40 years and older in the United States. Bethesda, MD National Eye Institute 2004; Available at http://www.nei.nih.gov/eyedata/pbd_tables.asp:

[2] Trick LR (1987). Age-related alterations in retinal function.. Doc Ophthalmol.

[3] Bonnel S, Mohand-Said S, Sahel JA (2003). The aging of the retina.. Exp Gerontol.

[4] Jackson GR, Owsley C, Cordle EP, Finley CD (1998). Aging and scotopic sensitivity.. Vision Res.

[5] Owsley C, Sekuler R, Siemsen D (1983). Contrast sensitivity throughout adulthood.. Vision Res.

[6] Sloane ME, Owsley C, Jackson CA (1988). Aging and luminance-adaptation effects on spatial contrast sensitivity.. J Opt Soc Am A.

[7] Sloane ME, Owsley C, Alvarez SL (1988). Aging, senile miosis and spatial contrast sensitivity at low luminance.. Vision Res.

[8] Jackson GR, Owsley C, McGwin G (1999). Aging and dark adaptation.. Vision Res.

[9] Birch DG, Anderson JL (1992). Standardized full-field electroretinography. Normal values and their variation with age.. Arch Ophthalmol.

[10] Jackson GR, Ortega J, Girkin C, Rosenstiel CE, Owsley C (2002). Aging-related changes in the multifocal electroretinogram.. J Opt Soc Am A Opt Image Sci Vis.

[11] Kurtenbach A, Weiss M (2002). Effect of aging on multifocal oscillatory potentials.. J Opt Soc Am A Opt Image Sci Vis.

[12] Weleber RG (1981). The effect of age on human cone and rod ganzfeld electroretinograms.. Invest Ophthalmol Vis Sci.

[13] Gerth C, Garcia SM, Ma L, Keltner JL, Werner JS (2002). Multifocal electroretinogram: age-related changes for different luminance levels.. Graefes Arch Clin Exp Ophthalmol.

[14] Ehrlich R, Kheradiya NS, Winston DM, Moore DB, Wirostko B, Harris A (2009). Age-related ocular vascular changes.. Graefes Arch Clin Exp Ophthalmol.

[15] Kneser M, Kohlmann T, Pokorny J, Tost F (2009). Age related decline of microvascular regulation measured in healthy individuals by retinal dynamic vessel analysis.. Med Sci Monit.

[16] Anderson DH, Radeke MJ, Gallo NB, Chapin EA, Johnson PT, Curletti CR, Hancox LS, Hu J, Ebright JN, Malek G, Hauser MA, Rickman CB, Bok D, Hageman GS, Johnson LV (2010). The pivotal role of the complement system in aging and age-related macular degeneration: hypothesis re-visited.. Prog Retin Eye Res.

[17] Boulton M, Roanowska M, Wess T (2004). Ageing of the retinal pigment epithelium: implications for transplantation.. Graefes Arch Clin Exp Ophthalmol.

[18] Ehrlich R, Harris A, Kheradiya NS, Winston DM, Ciulla TA, Wirostko B (2008). Age-related macular degeneration and the aging eye.. Clin Interv Aging.

[19] Johansen JS, Pedersen AN, Schroll M, Jorgensen T, Pedersen BK, Bruunsgaard H (2008). High serum YKL-40 level in a cohort of octogenarians is associated with increased risk of all-cause mortality.. Clin Exp Immunol.

[20] Chan-Ling T, Hughes S, Baxter L, Rosinova E, McGregor I, Morcos Y (2007). van NP, and Hu P. Inflammation and breakdown of the blood-retinal barrier during “physiological aging” in the rat retina: a model for CNS aging.. Microcirculation.

[21] Hollyfield JG, Bonilha VL, Rayborn ME, Yang X, Shadrach KG, Lu L, Ufret RL, Salomon RG, Perez VL (2008). Oxidative damage-induced inflammation initiates age-related macular degeneration.. Nat Med.

[22] Roy S, Tonkiss J, Roy S (2010). Aging increases retinal vascular lesions characteristic of early diabetic retinopathy.. Biogerontology.

[23] Parapuram SK, Cojocaru RI, Chang JR, Khanna R, Brooks M, Othman M, Zareparsi S, Khan NW, Gotoh N, Cogliati T, Swaroop A (2010). Distinct signature of altered homeostasis in aging rod photoreceptors: implications for retinal diseases.. PLoS ONE.

[24] Artola A, Kamal A, Ramakers GM, Gardoni F, Di LM, Biessels GJ, Cattabeni F, Gispen WH (2002). Synaptic plasticity in the diabetic brain: advanced aging?. Prog Brain Res.

[25] Artola A, Kamal A, Ramakers GM, Biessels GJ, Gispen WH (2005). Diabetes mellitus concomitantly facilitates the induction of long-term depression and inhibits that of long-term potentiation in hippocampus.. Eur J Neurosci.

[26] Artola A (2008). Diabetes-, stress- and ageing-related changes in synaptic plasticity in hippocampus and neocortex–the same metaplastic process?. Eur J Pharmacol.

[27] Trougakos IP, Poulakou M, Stathatos M, Chalikia A, Melidonis A, Gonos ES (2002). Serum levels of the senescence biomarker clusterin/apolipoprotein J increase significantly in diabetes type II and during development of coronary heart disease or at myocardial infarction.. Exp Gerontol.

[28] Amos AF, McCarty DJ, Zimmet P (1997). The rising global burden of diabetes and its complications: estimates and projections to the year 2010.. Diabet Med.

[29] Nooyens AC, Baan CA, Spijkerman AM, Verschuren WM (2010). Type 2 diabetes mellitus and cognitive decline in middle-aged men and women - The Doetinchem Cohort Study.. Diabetes Care.

[30] Bourdel-Marchasson I, Lapre E, Laksir H, Puget E (2010). Insulin resistance, diabetes and cognitive function: consequences for preventative strategies.. Diabetes Metab.

[31] Tomar RH, Lee S, Wu SY, Klein R, Klein BE, Moss SE, Fryback DG, Tollios JL, Sainfort F (1998). Disease progression and cost of insulin dependent diabetes mellitus: development and application of a simulation model.. J Soc Health Syst.

[32] Klein R, Marino EK, Kuller LH, Polak JF, Tracy RP, Gottdiener JS, Burke GL, Hubbard LD, Boineau R (2002). The relation of atherosclerotic cardiovascular disease to retinopathy in people with diabetes in the Cardiovascular Health Study.. Br J Ophthalmol.

[33] Klein R, Klein BE, Moss SE, Cruickshanks KJ (1998). The Wisconsin Epidemiologic Study of Diabetic Retinopathy: XVII. The 14-year incidence and progression of diabetic retinopathy and associated risk factors in type 1 diabetes.. Ophthalmology.

[34] Aiello LM (1998). Preserving human vision: eliminating blindness from diabetes mellitus.. J Am Optom Assoc.

[35] Davis MD, Fisher MR, Gangnon RE, Barton F, Aiello LM, Chew EY, Ferris FL, Knatterud GL (1998). Risk factors for high-risk proliferative diabetic retinopathy and severe visual loss: Early Treatment Diabetic Retinopathy Study Report #18.. Invest Ophthalmol Vis Sci.

[36] Frost-Larsen K, Larsen HW, Simonsen SE (1981). Value of electroretinography and dark adaptation as prognostic tools in diabetic retinopathy.. Dev Ophthalmol.

[37] Hyvärinen L, Laurinen P, Rovamo J (1983). Contrast sensitivity in evaluation of visual impairment due to diabetes.. Acta Ophthalmol (Copenh).

[38] Sokol S, Moskowitz A, Skarf B, Evans R, Molitch M, Senior B (1985). Contrast sensitivity in diabetics with and without background retinopathy.. Arch Ophthalmol.

[39] Di Leo MA, Caputo S, Falsini B, Porciatti V, Greco AV, Ghirlanda G (1994). Presence and further development of retinal dysfunction after 3-year follow up in IDDM patients without angiographically documented vasculopathy.. Diabetologia.

[40] Spaide RF, Fisher YL (2006). Intravitreal bevacizumab (Avastin) treatment of proliferative diabetic retinopathy complicated by vitreous hemorrhage.. Retina.

[41] Di Leo MA, Caputo S, Falsini B, Porciatti V, Minnella A, Greco AV, Ghirlanda G (1992). Nonselective loss of contrast sensitivity in visual system testing in early type I diabetes.. Diabetes Care.

[42] Dosso AA, Bonvin ER, Morel Y, Golay A, Assal JP, Leuenberger PM (1996). Risk factors associated with contrast sensitivity loss in diabetic patients.. Graefes Arch Clin Exp Ophthalmol.

[43] Barber AJ, Antonetti DA, Gardner TW (2000). Altered expression of retinal occludin and glial fibrillary acidic protein in experimental diabetes. The Penn State Retina Research Group.. Invest Ophthalmol Vis Sci.

[44] Antonetti DA, Barber AJ, Bronson SK, Freeman WM, Gardner TW, Jefferson LS, Kester M, Kimball SR, Krady JK, Lanoue KF, Norbury CC, Quinn PG, Sandirasegarane L, Simpson IA (2006). Diabetic retinopathy: seeing beyond glucose-induced microvascular disease.. Diabetes.

[45] Brucklacher RM, Patel KM, VanGuilder HD, Bixler GV, Barber AJ, Antonetti DA, Lin CM, Lanoue KF, Gardner TW, Bronson SK, Freeman WM (2008). Whole genome assessment of the retinal response to diabetes reveals a progressive neurovascular inflammatory response.. BMC Med Genomics.

[46] Freeman WM, Bixler GV, Brucklacher RM, Lin CM, Patel KM, Vanguilder HD, Lanoue KF, Kimball SR, Barber AJ, Antonetti DA, Gardner TW, Bronson SK (2010). A multistep validation process of biomarkers for preclinical drug development.. Pharmacogenomics J.

[47] VanGuilder HD, Brucklacher RM, Patel K, Ellis RW, Freeman WM, Barber AJ (2008). Diabetes downregulates presynaptic proteins and reduces basal synapsin I phosphorylation in rat retina.. Eur J Neurosci.

[48] Freeman WM, Bixler GV, Brucklacher RM, Walsh E, Kimball SR, Jefferson LS, Bronson SK (2009). Transcriptomic comparison of the retina in two mouse models of diabetes.. J Ocul Biol Dis Infor.

[49] VanGuilder HD, Bixler GV, Kutzler L, Brucklacher RM, Bronson SK, Kimball SR, Freeman WM (2011). Multi-modal proteomic analysis of retinal protein expression alterations in a rat model of diabetic retinopathy.. PLoS ONE.

[50] Xu H, Chen M, Forrester JV (2009). Para-inflammation in the aging retina.. Prog Retin Eye Res.

[51] VanGuilder HD, Yan H, Farley JA, Sonntag WE, Freeman WM (2010). Aging alters the expression of neurotransmission-regulating proteins in the hippocampal synaptoproteome.. J Neurochem.

[52] Livak KJ, Schmittgen TD (2001). Analysis of relative gene expression data using real-time quantitative PCR and the 2(-Delta Delta C(T)). Methods.

[53] VanGuilder HD, Vrana KE, Freeman WM (2008). Twenty-five years of quantitative PCR for gene expression analysis.. Biotechniques.

[54] Benjamini Y, Hochberg Y (1995). Controlling the False Discovery Rate: a Practical and Powerful Approach to Multiple Testing.. J R Stat Soc Ser B Stat Meth.

[55] Ramasamy R, Vannucci SJ, Yan SS, Herold K, Yan SF, Schmidt AM (2005). Advanced glycation end products and RAGE: a common thread in aging, diabetes, neurodegeneration, and inflammation.. Glycobiology.

[56] Qiu C, Cotch MF, Sigurdsson S, Garcia M, Klein R, Jonasson F, Klein BE, Eiriksdottir G, Harris TB, van Buchem MA, Gudnason V, Launer LJ (2008). Retinal and cerebral microvascular signs and diabetes: the age, gene/environment susceptibility-Reykjavik study.. Diabetes.

[57] Medzhitov R (2008). Origin and physiological roles of inflammation.. Nature.

[58] Hagino N, Kobayashi S, Tsutsumi T, Horiuchi S, Nagai R, Setalo G, Dettrich E (2004). Vascular change of hippocampal capillary is associated with vascular change of retinal capillary in aging.. Brain Res Bull.

[59] Qiu C, Cotch MF, Sigurdsson S, Klein R, Jonasson F, Klein BE, Garcia M, Jonsson PV, Harris TB, Eiriksdottir G, Kjartansson O, van Buchem MA, Gudnason V, Launer LJ (2009). Microvascular lesions in the brain and retina: The age, gene/environment susceptibility-Reykjavik study.. Ann Neurol.

[60] Khalfaoui T, Lizard G, Beltaief O, Colin D, Ben HJ, Errais K, Ammous I, Zbiba W, Tounsi L, Zhioua R, Anane R, Ouertani-Meddeb A (2009). Immunohistochemical analysis of cellular adhesion molecules (ICAM-1, VCAM-1) and VEGF in fibrovascular membranes of patients with proliferative diabetic retinopathy: preliminary study.. Pathol Biol (Paris).

[61] Joussen AM, Murata T, Tsujikawa A, Kirchhof B, Bursell SE, Adamis AP (2001). Leukocyte-mediated endothelial cell injury and death in the diabetic retina.. Am J Pathol.

[62] Chen M, Muckersie E, Forrester JV, Xu H (2010). Immune activation in Retinal Aging: A Gene Expression Study.. Invest Ophthalmol Vis Sci.

[63] Miyamoto K, Khosrof S, Bursell SE, Rohan R, Murata T, Clermont AC, Aiello LP, Ogura Y, Adamis AP (1999). Prevention of leukostasis and vascular leakage in streptozotocin-induced diabetic retinopathy via intercellular adhesion molecule-1 inhibition.. Proc Natl Acad Sci USA.

[64] Lam HC, Lee JK, Lu CC, Chu CH, Chuang MJ, Wang MC (2003). Role of endothelin in diabetic retinopathy.. Curr Vasc Pharmacol.

[65] Rattner A, Nathans J (2005). The genomic response to retinal disease and injury: evidence for endothelin signaling from photoreceptors to glia.. J Neurosci.

[66] Chung HK, Choi SM, Ahn BO, Kwak HH, Kim JH, Kim WB (2005). Efficacy of troxerutin on streptozotocin-induced rat model in the early stage of diabetic retinopathy.. Arzneimittelforschung.

[67] Hammes HP, Lin J, Bretzel RG, Brownlee M, Breier G (1998). Upregulation of the vascular endothelial growth factor/vascular endothelial growth factor receptor system in experimental background diabetic retinopathy of the rat.. Diabetes.

[68] Gerhardinger C, Costa MB, Coulombe MC, Toth I, Hoehn T, Grosu P (2005). Expression of acute-phase response proteins in retinal Muller cells in diabetes.. Invest Ophthalmol Vis Sci.

[69] Gong Y, Wang X, Liu J, Shi L, Yin B, Peng X, Qiang B, Yuan J (2005). NSPc1, a mainly nuclear localized protein of novel PcG family members, has a transcription repression activity related to its PKC phosphorylation site at S183.. FEBS Lett.

[70] Wu X, Gong Y, Yue J, Qiang B, Yuan J, Peng X (2008). Cooperation between EZH2, NSPc1-mediated histone H2A ubiquitination and Dnmt1 in HOX gene silencing.. Nucleic Acids Res.

[71] Arning L, Kraus PH, Valentin S, Saft C, Andrich J, Epplen JT (2005). NR2A and NR2B receptor gene variations modify age at onset in Huntington disease.. Neurogenetics.

[72] Chen M, Muckersie E, Robertson M, Forrester JV, Xu H (2008). Up-regulation of complement factor B in retinal pigment epithelial cells is accompanied by complement activation in the aged retina.. Exp Eye Res.

[73] Khalyfa A, Chlon T, Qiang H, Agarwal N, Cooper NG (2007). Microarray reveals complement components are regulated in the serum-deprived rat retinal ganglion cell line.. Mol Vis.

[74] Rathcke CN, Vestergaard H (2009). YKL-40–an emerging biomarker in cardiovascular disease and diabetes.. Cardiovasc Diabetol.

[75] Nielsen AR, Erikstrup C, Johansen JS, Fischer CP, Plomgaard P, Krogh-Madsen R, Taudorf S, Lindegaard B, Pedersen BK (2008). Plasma YKL-40: a BMI-independent marker of type 2 diabetes.. Diabetes.

[76] Rathcke CN, Persson F, Tarnow L, Rossing P, Vestergaard H (2009). YKL-40, a marker of inflammation and endothelial dysfunction, is elevated in patients with type 1 diabetes and increases with levels of albuminuria.. Diabetes Care.

[77] O'Shea JJ, Pesu M, Borie DC, Changelian PS (2004). A new modality for immunosuppression: targeting the JAK/STAT pathway.. Nat Rev Drug Discov.

[78] Srinivasan S, Leeman SE, Amar S (2010). Beneficial dysregulation of the time course of inflammatory mediators in lipopolysaccharide-induced tumor necrosis factor alpha factor-deficient mice.. Clin Vaccine Immunol.

[79] Ebihara N, Nakayama M, Tokura T, Iwatsu M, Ushio H, Murakami A (2009). Proinflammatory effect of TWEAK/Fn14 interaction in human retinal pigment epithelial cells.. Curr Eye Res.

[80] Ho DH, Vu H, Brown SA, Donohue PJ, Hanscom HN, Winkles JA (2004). Soluble tumor necrosis factor-like weak inducer of apoptosis overexpression in HEK293 cells promotes tumor growth and angiogenesis in athymic nude mice.. Cancer Res.

[81] Katakami N, Kaneto H, Yamasaki Y, Matsuhisa M (2008). Increased serum pigment epithelium-derived factor levels in type 1 diabetic patients with diabetic retinopathy.. Diabetes Res Clin Pract.

[82] Dawson DW, Volpert OV, Gillis P, Crawford SE, Xu H, Benedict W, Bouck NP (1999). Pigment epithelium-derived factor: a potent inhibitor of angiogenesis.. Science.

[83] Sheikpranbabu S, Haribalaganesh R, Lee KJ, Gurunathan S (2010). Pigment epithelium-derived factor inhibits advanced glycation end products-induced retinal vascular permeability.. Biochimie.

[84] Gao BB, Clermont A, Rook S, Fonda SJ, Srinivasan VJ, Wojtkowski M, Fujimoto JG, Avery RL, Arrigg PG, Bursell SE, Aiello LP, Feener EP (2007). Extracellular carbonic anhydrase mediates hemorrhagic retinal and cerebral vascular permeability through prekallikrein activation.. Nat Med.

[85] Paulus YM, Gariano RF (2009). Diabetic retinopathy: a growing concern in an aging population.. Geriatrics.

